# Supplementary material for: A Reconstructed Human Melanoma-in-Skin Model to Study Immune Modulatory and Angiogenic Mechanisms Facilitating Initial Melanoma Growth and Invasion
Source: Cancers (Basel). 2023 May 20;15(10):2849. doi: 10.3390/cancers15102849 (PMC10216824; doi:10.3390/cancers15102849)
Supplement: Supplementary file 1 [file cancers-15-02849-s001.zip › Appendix Supplementary figures.pdf]

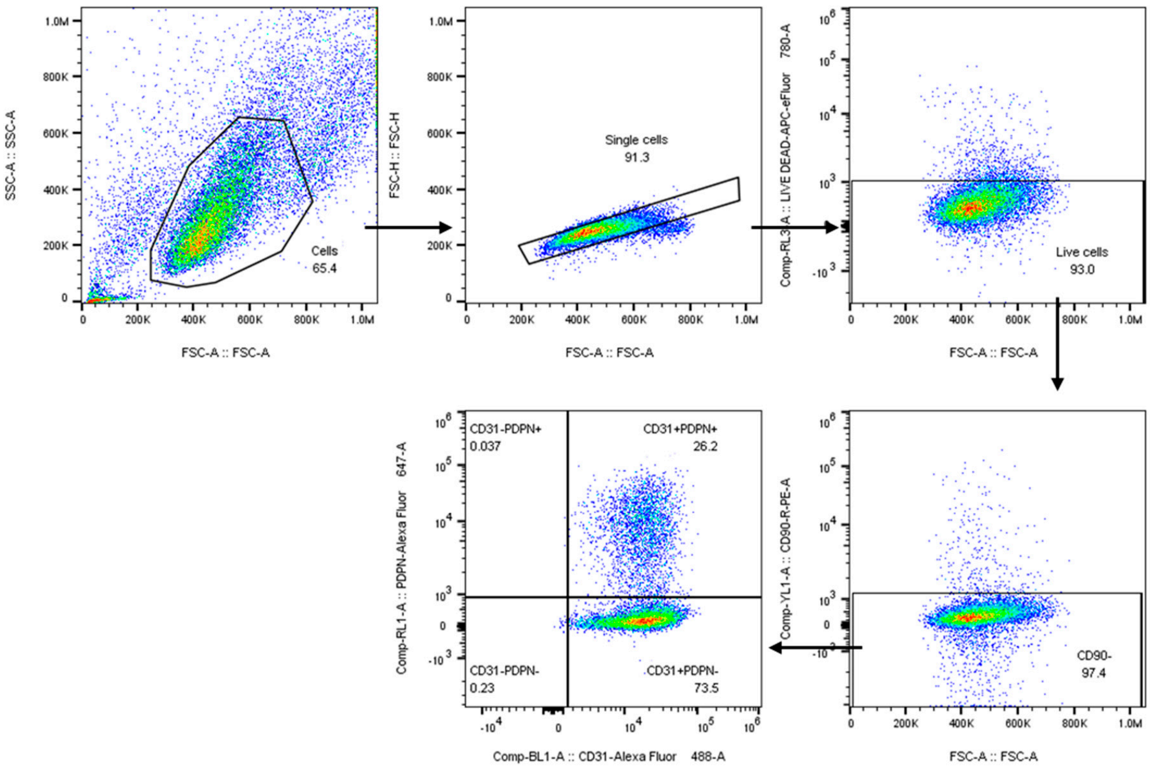

Figure S1. Gating strategy to analyze dermal endothelial cells (ECs).

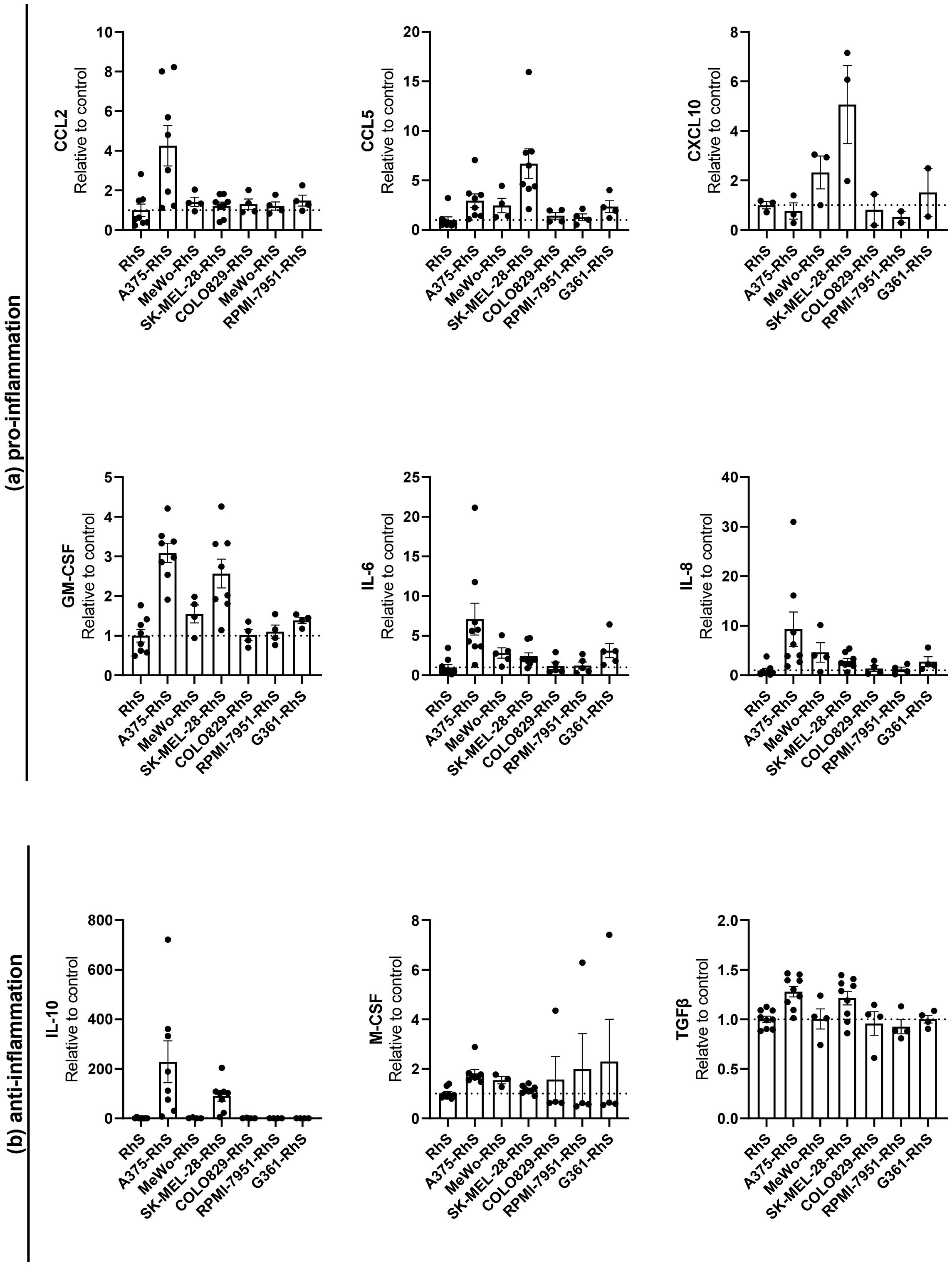

Figure S2. Cytokine secretion in the supernatants from either RhS or Mel-RhS constructed with A375, MeWo, SK-MEL-28, COLO829, RPMI-7951, G361 cells.

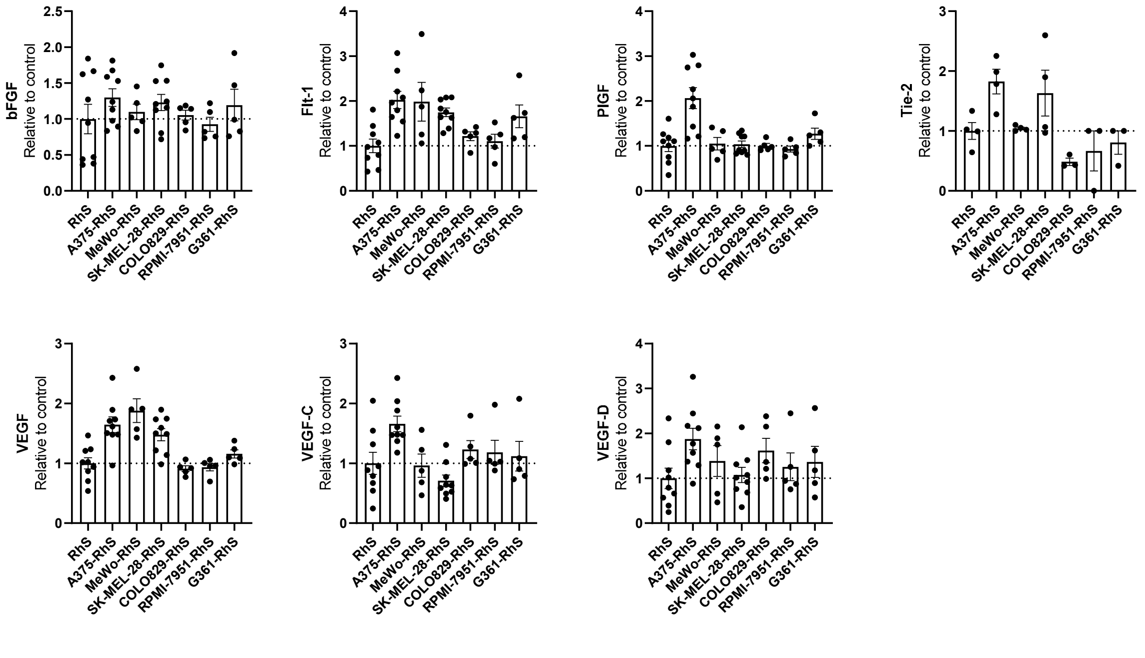

Figure S3. Levels of pro-angiogenic factors in supernatants from either RhS or Mel-RhS constructed with A375, MeWo, SK-MEL-28, COLO829, RPMI-7951, G361 cells.

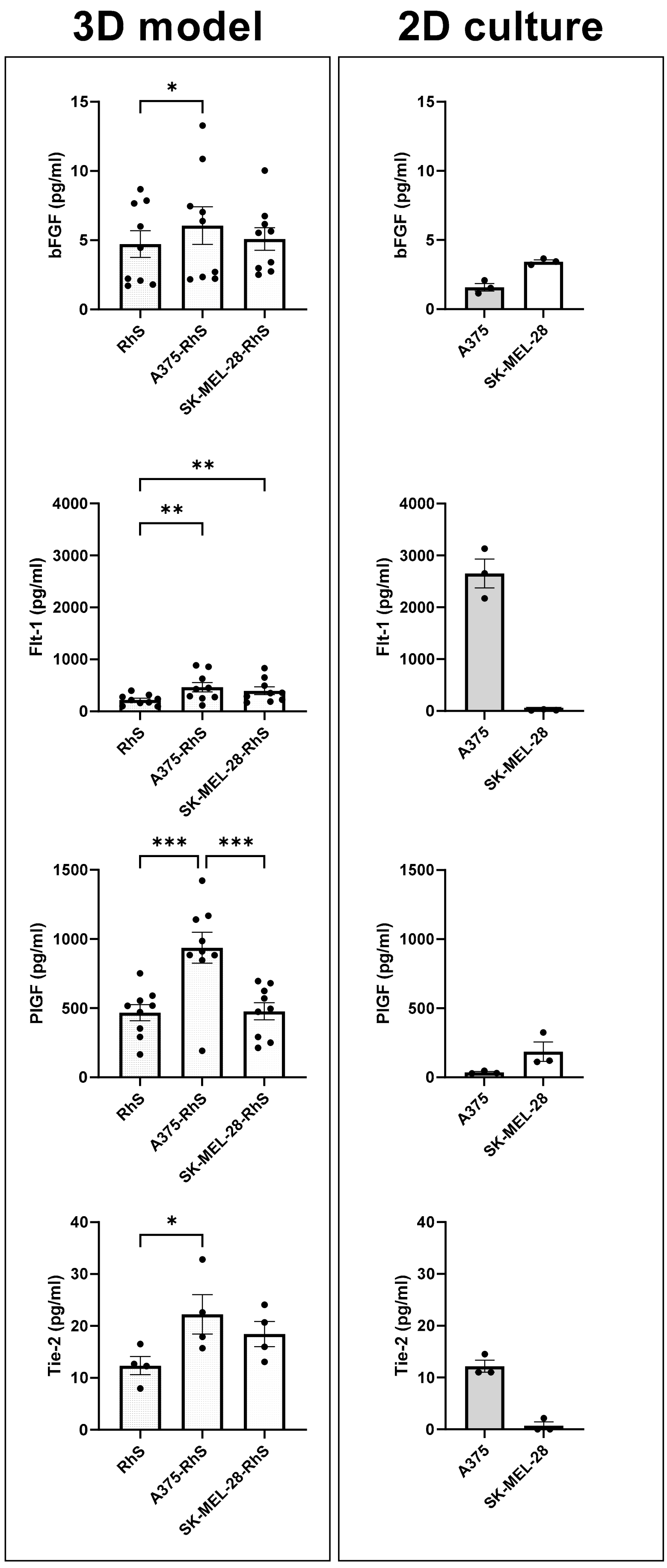
3D model
2D culture
bFGF (pg/ml)
Flt-1 (pg/ml)
PlGF (pg/ml)
Tie-2 (pg/ml)
RhS
A375-RhS
SK-MEL-28-RhS
A375
SK-MEL-28
*
**
***

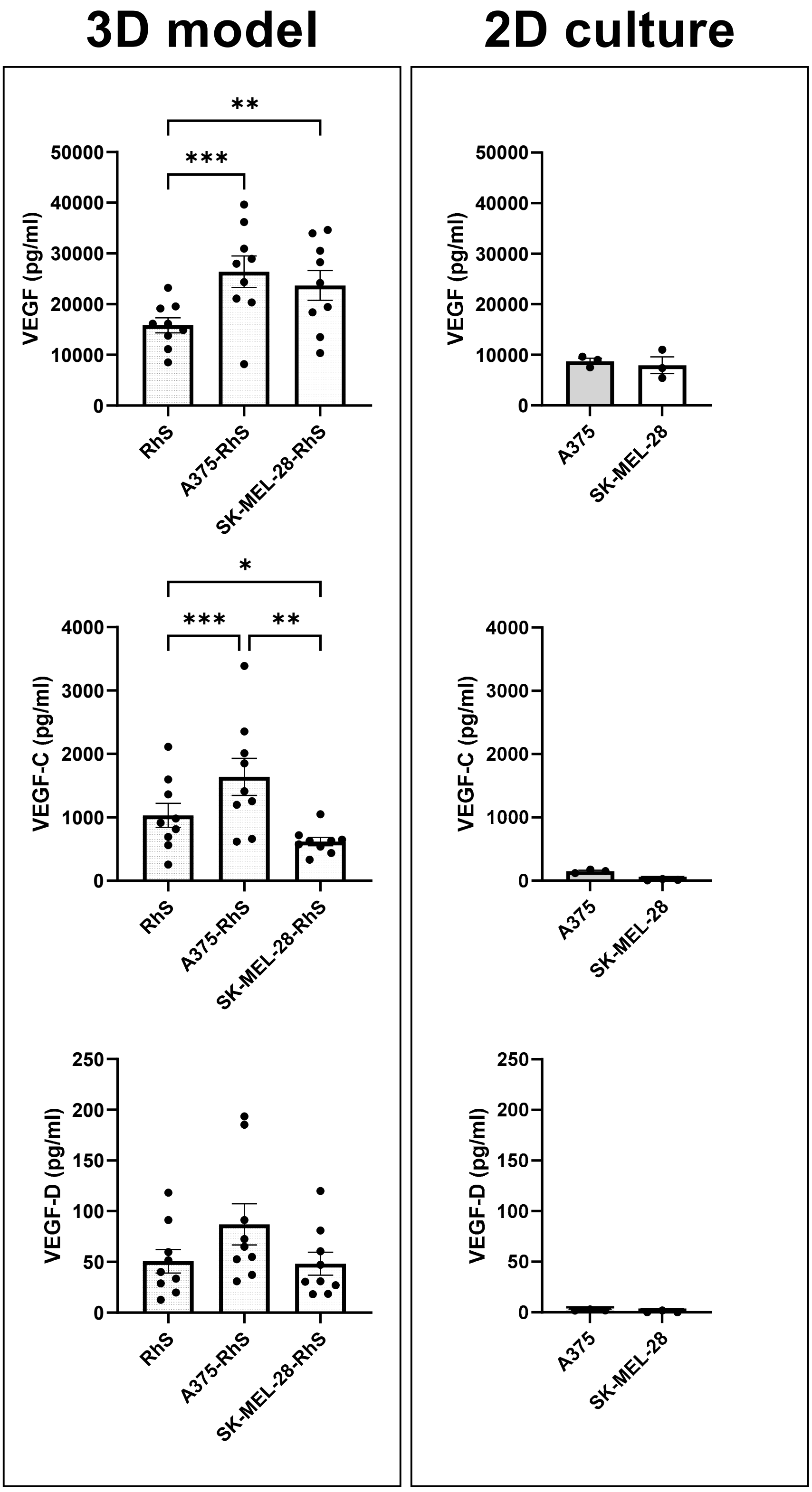

Figure S4. Release of angiogenic factors in cultures supernatants from the 3D models (right panel) and from confluent 2D melanoma cell monolayers (right panel).

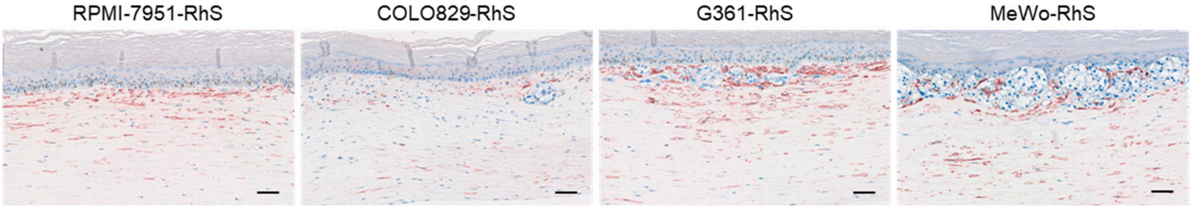

Figure S5. Mel-RhS constructed with either RPMI-7951, COLO829, G361, or MeWo cells were cultured for 4 weeks at the air-liquid interface and stained for α-SMA.

Table S1. Cytokine secretion in the supernatant of the skin (RhS) and melanoma (Mel-RhS) models constructed with the different melanoma cell lines (A375, COLO829, G361, MeWo, RPMI-7951, and SK-MEL-28).

Cytokine	RhS	A375-RhS	COLO829-RhS	G361-RhS	MeWo-RhS	RPMI-7951-RhS	SK-MEL-28-RhS
**CCL2**	14506 ± 4575	84492 ± 39764	6568 ± 415.1	7488 ± 91.83	7242 ± 162.2	6151 ± 215.5	13667 ± 3817
**CCL5**	37.27 ± 12.21	86.66 ± 17.54	55.86 ± 19.81	81.71 ± 22.65	90.16 ± 30.25	42.13 ± 9.477	183.7 ± 25.54
**CXCL10**	65.75 ± 28.15	69.25 ± 29.59	82.98 ± 36.85	113.7 ± 71.38	184.7 ± 80.83	34.7 ± 21.58	353.5 ± 137.4
**IL-6**	3886 ± 1395	20962 ± 9180	3712 ± 812.7	12153 ± 3559	11837 ± 3594	3528 ± 250.4	7930 ± 2728
**IL-8**	3771 ± 1594	19649 ± 4738	2968 ± 701.7	8070 ± 3461	8886 ± 738.6	2461 ± 366.3	8927 ± 3673
**IL-10**	1.657 ± 1.514	149.7 ± 44.59	0.7588 ± 0.7588	0	0.9325 ± 0.9325	0	61.39 ± 9.154
**GM-CSF**	50.2 ± 8.126	148.5 ± 23.68	55.61 ± 18.79	76.6 ± 24.72	78.07 ± 23.1	52.29 ± 11.39	117.3 ± 16
**M-CSF**	667.2 ± 157.8	1158 ± 227.2	1037 ± 77.72	1213 ± 54.38	1182 ± 87.5	917.2 ± 32.54	820.2 ± 162.7
**TGFβ**	495.5 ± 30.05	688.3 ± 64.73	456.7 ± 46.05	482.9 ± 23.68	478.8 ± 30.48	442.7 ± 15.96	618.7 ± 28.93
**bFGF**	4.714 ± 0.9627	6.053 ± 1.361	7.158 ± 0.6177	7.614 ± 0.4998	7.395 ± 0.6866	6.394 ± 1.067	5.082 ± 0.8192
**Flt-1**	221.2 ± 33.44	464.7 ± 90.62	272.5 ± 57.05	391.8 ± 122.1	459.8 ± 169.6	248 ± 67.23	395.7 ± 74.89
**PlGF**	468 ± 58.2	937.3 ± 111.9	367.5 ± 65.27	474 ± 114.7	384.6 ± 95.64	344.9 ± 73.49	477.3 ± 61.8
**Tie-2**	12.36 ± 1.751	22.26 ± 3.799	2.924 ± 1.502	2.424 ± 1.197	1.447 ± 0.9494	0	18.46 ± 2.441
**VEGF**	15838 ± 1493	26412 ± 3121	11781 ± 1005	15459 ± 2439	25497 ± 5001	12046 ± 1114	23703 ± 2935
**VEGF-C**	1032 ± 189.9	1639 ± 292.2	945.4 ± 229.6	778.5 ± 150.1	710.6 ± 164.1	850.3 ± 161.1	617.5 ± 66.57
**VEGF-D**	50.64 ± 11.54	87.07 ± 20.26	42.48 ± 7.53	33.36 ± 4.92	35.87 ± 10.05	31.74 ± 5.264	48.21 ± 11.3

Table S2. Pearson correlation between cytokines secreted in culture supernatants from RhS and SK-MEL-28-RhS and marker expression on conditioned monocytes.

p value	CD1a+	CD1a-CD14-	CD14+	BDCA3+	BDCA3-CD14-	BDCA3-CD14+	BDCA3+CD14-	BDCA3+CD14+	CD80+	CD80 MFI	CD163+	CD163 MFI	CD16+	CD16 MFI	CD14+CD163+CD16+	PD-L1 MFI	PD-L1+	PD-L2 MFI	PD-L2+
CCL2	0.171573	0.688114	0.430910	0.755713	0.508506	0.526741	0.632399	0.862817	0.820723	0.706118	0.593804	0.201566	0.808425	0.256283	0.388472	0.398332	0.535006	0.188975	0.514280
CCL22	0.991597	0.638654	0.731774	0.203271	0.705103	0.616225	0.793134	0.198237	0.971637	0.296806	0.803854	0.806703	0.766872	0.927275	0.739749	0.613963	0.906270	0.464167	0.897886
CXCL10	0.214935	0.378734	0.276398	0.710167	0.246233	0.471091	0.277815	0.253440	0.224824	0.395681	0.184481	0.684193	0.183553	0.519849	0.245382	0.164434	0.511369	0.547993	0.469971
IL-6	0.062034	0.487428	0.240870	0.270529	0.309363	0.670713	0.444741	0.208097	0.718455	0.965267	0.375904	0.157113	0.584420	0.172379	0.216676	0.080388	0.431894	0.091645	0.405030
IL-8	0.022036	0.164483	0.064580	0.392551	0.118266	0.262112	0.164318	0.227577	0.396799	0.751583	0.123708	0.042338	0.222381	0.039682	0.061753	0.036697	0.270138	0.050721	0.280892
IL-10	0.032359	0.044977	0.024000	0.171094	0.025510	0.280466	0.078584	0.011478	0.074061	0.442844	0.022445	0.062965	0.023281	0.056571	0.020288	0.020130	0.272960	0.053524	0.296346
GM-CSF	0.449503	0.135376	0.172404	0.648545	0.204314	0.100909	0.161138	0.955596	0.021766	0.968726	0.168173	0.103641	0.145229	0.125053	0.159199	0.462718	0.250454	0.234931	0.283769
M-CSF	0.034442	0.067172	0.033789	0.894102	0.092521	0.152229	0.086379	0.299101	0.469543	0.769497	0.059959	0.124938	0.094943	0.102735	0.041577	0.166302	0.315194	0.170407	0.339928
TGFβ	0.002745	0.004288	0.001126	0.751666	0.010261	0.008632	0.003872	0.332553	0.038951	0.378559	0.004276	0.007781	0.009997	0.002956	0.001601	0.054487	0.049802	0.048747	0.072369
VEGF	0.774302	0.957164	0.983065	0.028074	0.563413	0.076326	0.820349	0.034507	0.125375	0.117352	0.832265	0.875228	0.710526	0.672113	0.871149	0.012313	0.746955	0.581788	0.504836

Pearson *r* value	CD1a+	CD1a-CD14-	CD14+	BDCA3+	BDCA3-CD14-	BDCA3-CD14+	BDCA3+CD14-	BDCA3+CD14+	CD80+	CD80 MFI	CD163+	CD163 MFI	CD16+	CD16 MFI	CD14+CD163+CD16+	PD-L1 MFI	PD-L1+	PD-L2 MFI	PD-L2+
CCL2	-0.30991	-0.09311	0.18156	0.07222	-0.15278	0.14634	-0.11085	0.04015	0.05264	-0.08748	0.12350	0.29042	0.05632	0.25934	0.19846	0.19445	0.14346	0.29834	0.15073
CCL22	0.00245	0.10883	-0.07955	-0.28937	0.08780	0.11611	-0.06090	-0.29248	-0.00826	-0.23898	-0.05768	-0.05683	-0.06883	-0.02121	-0.07710	-0.11685	0.02736	-0.16893	0.02982
CXCL10	-0.28236	-0.20248	0.24900	0.08623	-0.26470	0.16636	-0.24829	0.26084	0.27660	0.19552	0.30126	0.09434	0.30187	0.14876	0.26516	0.31489	0.15176	0.13897	0.16677
IL-6	-0.41406	-0.16037	0.26761	0.25197	-0.23303	0.09859	-0.17624	0.28644	0.08366	0.01012	0.20366	0.32015	0.12662	0.30936	0.28133	0.39014	0.18118	0.37744	0.19175
IL-8	-0.49656	-0.31485	0.41045	0.19679	-0.35142	0.25629	-0.31497	0.27503	0.19507	-0.07348	0.34663	0.44673	0.27801	0.45199	0.41447	0.45824	0.25216	0.43164	0.24676
IL-10	-0.46808	-0.44175	0.49042	0.31024	-0.48597	0.24697	-0.39230	0.54017	0.39788	0.17697	0.49525	0.41273	0.49262	0.42221	0.50240	0.50294	0.25073	0.42702	0.23920
GM-CSF	0.17444	0.33687	-0.30934	0.10565	0.28873	-0.36782	0.31724	0.01294	-0.49744	-0.00911	-0.31226	-0.36511	-0.32907	-0.34548	-0.31864	-0.16947	-0.26243	-0.27090	-0.24533
M-CSF	-0.46323	-0.40688	0.46473	0.03094	-0.37650	0.32376	-0.38323	0.23788	0.16693	-0.06804	0.41709	0.34558	0.37394	0.36600	0.44821	0.31357	0.23031	0.31071	0.21912
TGFβ	-0.61949	-0.59682	0.66020	-0.07346	-0.54713	0.55759	-0.60216	0.22240	0.45348	-0.20255	0.59697	0.56372	0.54873	0.61585	0.64481	0.42548	0.43320	0.43501	0.40003
VEGF	-0.15163	0.02857	0.01129	0.85988	-0.30006	-0.76504	0.12035	0.84423	0.69497	0.70546	0.11230	0.08337	0.19547	0.22225	0.08611	0.90798	0.17034	0.28666	0.34364

Table S3. Pearson correlation between cytokines secreted in culture supernatants from RhS and A375-RhS and marker expression on conditioned monocytes.

p value	CD1a+	CD1a-CD14-	CD14+	BDCA3+	BDCA3-CD14-	BDCA3-CD14+	BDCA3+CD14-	BDCA3+CD14+	CD80+	CD80 MFI	CD163+	CD163 MFI	CD16+	CD16 MFI	CD14+CD163+CD16+	PD-L1+	PD-L1 MFI	PD-L2+	PD-L2 MFI
IL-6	0.566340	0.497208	0.705485	0.163711	0.226820	0.940852	0.970656	0.253647	0.329764	0.311409	0.787161	0.979386	0.681586	0.506113	0.526620	0.149104	0.074669	0.172146	0.032416
IL-8	0.599937	0.564227	0.739890	0.059851	0.199063	0.980599	0.939462	0.182013	0.462123	0.388216	0.549260	0.763992	0.697427	0.697846	0.694804	0.065855	0.024026	0.167916	0.017167
CXCL10	0.139219	0.751087	0.534977	0.382039	0.832484	0.718230	0.953138	0.585787	0.869414	0.437987	0.924267	0.561376	0.118981	0.180866	0.097945	0.259120	0.191265	0.297249	0.055466
CCL2	0.693415	0.373143	0.567059	0.004238	0.209313	0.568373	0.175441	0.003255	0.132623	0.061141	0.837639	0.912877	0.501435	0.246335	0.450047	0.216393	0.020589	0.588459	0.214266
CCL5	0.221260	0.975944	0.754002	0.086790	0.488875	0.750294	0.880305	0.269979	0.727195	0.366598	0.737691	0.930032	0.257846	0.436158	0.306669	0.066600	0.027180	0.192536	0.008709
VEGF	0.315963	0.759816	0.627339	0.610769	0.799957	0.964407	0.532920	0.948147	0.867101	0.860702	0.696745	0.788031	0.100095	0.071798	0.075714	0.282859	0.322844	0.246683	0.066847
IL-10	0.946256	0.982846	0.938422	0.932465	0.771562	0.702597	0.997989	0.758208	0.896934	0.669116	0.305868	0.168834	0.816738	0.575086	0.417391	0.187821	0.707109	0.544127	0.810669
TGFβ	0.115299	0.888780	0.609571	0.020666	0.699310	0.299653	0.407207	0.041224	0.578889	0.080649	0.521627	0.300938	0.287164	0.627054	0.234675	0.408574	0.031262	0.578935	0.045041
M-CSF	0.411972	0.413290	0.688034	0.043791	0.347541	0.466835	0.005039	0.013596	0.065102	0.015055	0.544995	0.740748	0.296428	0.018228	0.177926	0.244427	0.110815	0.623570	0.475130

Pearson *r* value	CD1a+	CD1a-CD14-	CD14+	BDCA3+	BDCA3-CD14-	BDCA3-CD14+	BDCA3+CD14-	BDCA3+CD14+	CD80+	CD80 MFI	CD163+	CD163 MFI	CD16+	CD16 MFI	CD14+CD163+CD16+	PD-L1+	PD-L1 MFI	PD-L2+	PD-L2 MFI
IL-6	0.240385	-0.282885	0.159773	0.543634	-0.481683	0.031567	-0.015653	0.458116	0.397283	0.411271	0.114511	-0.010995	-0.173257	-0.277293	-0.264554	0.559732	0.660399	0.534693	0.749134
IL-8	0.220400	-0.241656	0.140566	0.686885	-0.507633	0.010348	0.032309	0.524527	0.305310	0.354978	0.250701	0.127238	-0.164305	-0.164069	-0.165784	0.675732	0.774536	0.539146	0.799806
CXCL10	0.571124	0.134362	-0.259416	0.359306	-0.089824	-0.152631	-0.025004	0.228770	0.069873	0.321136	-0.040435	-0.243372	-0.595941	-0.525694	-0.624416	0.453465	0.515260	0.422333	0.695459
CCL2	0.166567	-0.365594	0.239953	0.876953	-0.497843	-0.239165	0.531264	0.887632	0.578976	0.684434	-0.087031	0.046533	0.280226	0.464409	0.313185	0.491226	0.786540	0.227185	0.493201
CCL5	0.486743	0.012831	-0.132750	0.640958	-0.288154	-0.134800	0.064012	0.444381	0.147627	0.370264	0.141787	-0.037351	-0.454543	-0.322350	-0.414947	0.674391	0.764428	0.514005	0.842127
VEGF	0.407765	0.129540	-0.204372	0.214037	-0.107515	0.018988	-0.260678	0.027669	-0.071119	0.074568	0.164689	-0.114035	-0.621354	-0.665272	-0.658653	0.433838	0.402513	0.464107	0.673948
IL-10	-0.028679	-0.009149	0.032865	0.036050	-0.123071	0.161396	-0.001072	-0.130427	-0.055080	-0.180344	0.415570	0.538174	0.098372	0.235145	0.334930	0.518682	0.158861	0.253824	0.101675
TGFβ	-0.600705	-0.059457	0.214739	-0.786257	0.163245	0.420437	-0.341857	-0.726510	-0.232875	-0.650595	0.267639	0.419427	0.430367	0.204537	0.474642	-0.340923	-0.752367	-0.232847	-0.717625
M-CSF	0.338607	-0.337710	0.169607	0.720482	-0.384078	-0.302260	0.869391	0.815562	0.677097	0.808844	-0.253295	0.140090	0.422982	0.795528	0.528701	0.466066	0.606625	0.206563	0.296921
