# Supplementary material for: A Reconstructed Human Melanoma-in-Skin Model to Study Immune Modulatory and Angiogenic Mechanisms Facilitating Initial Melanoma Growth and Invasion
Source: Cancers (Basel). 2023 May 20;15(10):2849. doi: 10.3390/cancers15102849 (PMC10216824; doi:10.3390/cancers15102849)
Supplement: Supplementary file 1 [file cancers-15-02849-s001.zip › Table S1.pdf]

Cytokine	RhS	A375-RhS	COLO829-RhS	G361-RhS	MeWo-RhS	RPMI-7951-RhS	SK-MEL-28-RhS
**CCL2**	14506 ± 4575	84492 ± 39764	6568 ± 415.1	7488 ± 91.83	7242 ± 162.2	6151 ± 215.5	13667 ± 3817
**CCL5**	37.27 ± 12.21	86.66 ± 17.54	55.86 ± 19.81	81.71 ± 22.65	90.16 ± 30.25	42.13 ± 9.477	183.7 ± 25.54
**CXCL10**	65.75 ± 28.15	69.25 ± 29.59	82.98 ± 36.85	113.7 ± 71.38	184.7 ± 80.83	34.7 ± 21.58	353.5 ± 137.4
**IL-6**	3886 ± 1395	20962 ± 9180	3712 ± 812.7	12153 ± 3559	11837 ± 3594	3528 ± 250.4	7930 ± 2728
**IL-8**	3771 ± 1594	19649 ± 4738	2968 ± 701.7	8070 ± 3461	8886 ± 738.6	2461 ± 366.3	8927 ± 3673
**IL-10**	1.657 ± 1.514	149.7 ± 44.59	0.7588 ± 0.7588	0	0.9325 ± 0.9325	0	61.39 ± 9.154
**GM-CSF**	50.2 ± 8.126	148.5 ± 23.68	55.61 ± 18.79	76.6 ± 24.72	78.07 ± 23.1	52.29 ± 11.39	117.3 ± 16
**M-CSF**	667.2 ± 157.8	1158 ± 227.2	1037 ± 77.72	1213 ± 54.38	1182 ± 87.5	917.2 ± 32.54	820.2 ± 162.7
**TGFβ**	495.5 ± 30.05	688.3 ± 64.73	456.7 ± 46.05	482.9 ± 23.68	478.8 ± 30.48	442.7 ± 15.96	618.7 ± 28.93
**bFGF**	4.714 ± 0.9627	6.053 ± 1.361	7.158 ± 0.6177	7.614 ± 0.4998	7.395 ± 0.6866	6.394 ± 1.067	5.082 ± 0.8192
**Flt-1**	221.2 ± 33.44	464.7 ± 90.62	272.5 ± 57.05	391.8 ± 122.1	459.8 ± 169.6	248 ± 67.23	395.7 ± 74.89
**PlGF**	468 ± 58.2	937.3 ± 111.9	367.5 ± 65.27	474 ± 114.7	384.6 ± 95.64	344.9 ± 73.49	477.3 ± 61.8
**Tie-2**	12.36 ± 1.751	22.26 ± 3.799	2.924 ± 1.502	2.424 ± 1.197	1.447 ± 0.9494	0	18.46 ± 2.441
**VEGF**	15838 ± 1493	26412 ± 3121	11781 ± 1005	15459 ± 2439	25497 ± 5001	12046 ± 1114	23703 ± 2935
**VEGF-C**	1032 ± 189.9	1639 ± 292.2	945.4 ± 229.6	778.5 ± 150.1	710.6 ± 164.1	850.3 ± 161.1	617.5 ± 66.57
**VEGF-D**	50.64 ± 11.54	87.07 ± 20.26	42.48 ± 7.53	33.36 ± 4.92	35.87 ± 10.05	31.74 ± 5.264	48.21 ± 11.3
