# Supplementary material for: A Reconstructed Human Melanoma-in-Skin Model to Study Immune Modulatory and Angiogenic Mechanisms Facilitating Initial Melanoma Growth and Invasion
Source: Cancers (Basel). 2023 May 20;15(10):2849. doi: 10.3390/cancers15102849 (PMC10216824; doi:10.3390/cancers15102849)
Supplement: Supplementary file 1 [file cancers-15-02849-s001.zip › Table S2.pdf]

p value	CD1a+	CD1a-CD14-	CD14+	BDCA3+	BDCA3-CD14-	BDCA3-CD14+	BDCA3+CD14-	BDCA3+CD14+	CD80+	CD80 MFI	CD163+	CD163 MFI	CD16+	CD16 MFI	CD14+CD163+CD16+	PD-L1 MFI	PD-L1+	PD-L2 MFI	PD-L2+
CCL2	0.171573	0.688114	0.430910	0.755713	0.508506	0.526741	0.632399	0.862817	0.820723	0.706118	0.593804	0.201566	0.808425	0.256283	0.388472	0.398332	0.535006	0.188975	0.514280
CCL22	0.991597	0.638654	0.731774	0.203271	0.705103	0.616225	0.793134	0.198237	0.971637	0.296806	0.803854	0.806703	0.766872	0.927275	0.739749	0.613963	0.906270	0.464167	0.897886
CXCL10	0.214935	0.378734	0.276398	0.710167	0.246233	0.471091	0.277815	0.253440	0.224824	0.395681	0.184481	0.684193	0.183553	0.519849	0.245382	0.164434	0.511369	0.547993	0.469971
IL-6	0.062034	0.487428	0.240870	0.270529	0.309363	0.670713	0.444741	0.208097	0.718455	0.965267	0.375904	0.157113	0.584420	0.172379	0.216676	0.080388	0.431894	0.091645	0.405030
IL-8	0.022036	0.164483	0.064580	0.392551	0.118266	0.262112	0.164318	0.227577	0.396799	0.751583	0.123708	0.042338	0.222381	0.039682	0.061753	0.036697	0.270138	0.050721	0.280892
IL-10	0.032359	0.044977	0.024000	0.171094	0.025510	0.280466	0.078584	0.011478	0.074061	0.442844	0.022445	0.062965	0.023281	0.056571	0.020288	0.020130	0.272960	0.053524	0.296346
GM-CSF	0.449503	0.135376	0.172404	0.648545	0.204314	0.100909	0.161138	0.955596	0.021766	0.968726	0.168173	0.103641	0.145229	0.125053	0.159199	0.462718	0.250454	0.234931	0.283769
M-CSF	0.034442	0.067172	0.033789	0.894102	0.092521	0.152229	0.086379	0.299101	0.469543	0.769497	0.059959	0.124938	0.094943	0.102735	0.041577	0.166302	0.315194	0.170407	0.339928
TGFβ	0.002745	0.004288	0.001126	0.751666	0.010261	0.008632	0.003872	0.332553	0.038951	0.378559	0.004276	0.007781	0.009997	0.002956	0.001601	0.054487	0.049802	0.048747	0.072369
VEGF	0.774302	0.957164	0.983065	0.028074	0.563413	0.076326	0.820349	0.034507	0.125375	0.117352	0.832265	0.875228	0.710526	0.672113	0.871149	0.012313	0.746955	0.581788	0.504836

Pearson *r* value	CD1a+	CD1a-CD14-	CD14+	BDCA3+	BDCA3-CD14-	BDCA3-CD14+	BDCA3+CD14-	BDCA3+CD14+	CD80+	CD80 MFI	CD163+	CD163 MFI	CD16+	CD16 MFI	CD14+CD163+CD16+	PD-L1 MFI	PD-L1+	PD-L2 MFI	PD-L2+
CCL2	-0.30991	-0.09311	0.18156	0.07222	-0.15278	0.14634	-0.11085	0.04015	0.05264	-0.08748	0.12350	0.29042	0.05632	0.25934	0.19846	0.19445	0.14346	0.29834	0.15073
CCL22	0.00245	0.10883	-0.07955	-0.28937	0.08780	0.11611	-0.06090	-0.29248	-0.00826	-0.23898	-0.05768	-0.05683	-0.06883	-0.02121	-0.07710	-0.11685	0.02736	-0.16893	0.02982
CXCL10	-0.28236	-0.20248	0.24900	0.08623	-0.26470	0.16636	-0.24829	0.26084	0.27660	0.19552	0.30126	0.09434	0.30187	0.14876	0.26516	0.31489	0.15176	0.13897	0.16677
IL-6	-0.41406	-0.16037	0.26761	0.25197	-0.23303	0.09859	-0.17624	0.28644	0.08366	0.01012	0.20366	0.32015	0.12662	0.30936	0.28133	0.39014	0.18118	0.37744	0.19175
IL-8	-0.49656	-0.31485	0.41045	0.19679	-0.35142	0.25629	-0.31497	0.27503	0.19507	-0.07348	0.34663	0.44673	0.27801	0.45199	0.41447	0.45824	0.25216	0.43164	0.24676
IL-10	-0.46808	-0.44175	0.49042	0.31024	-0.48597	0.24697	-0.39230	0.54017	0.39788	0.17697	0.49525	0.41273	0.49262	0.42221	0.50240	0.50294	0.25073	0.42702	0.23920
GM-CSF	0.17444	0.33687	-0.30934	0.10565	0.28873	-0.36782	0.31724	0.01294	-0.49744	-0.00911	-0.31226	-0.36511	-0.32907	-0.34548	-0.31864	-0.16947	-0.26243	-0.27090	-0.24533
M-CSF	-0.46323	-0.40688	0.46473	0.03094	-0.37650	0.32376	-0.38323	0.23788	0.16693	-0.06804	0.41709	0.34558	0.37394	0.36600	0.44821	0.31357	0.23031	0.31071	0.21912
TGFβ	-0.61949	-0.59682	0.66020	-0.07346	-0.54713	0.55759	-0.60216	0.22240	0.45348	-0.20255	0.59697	0.56372	0.54873	0.61585	0.64481	0.42548	0.43320	0.43501	0.40003
VEGF	-0.15163	0.02857	0.01129	0.85988	-0.30006	-0.76504	0.12035	0.84423	0.69497	0.70546	0.11230	0.08337	0.19547	0.22225	0.08611	0.90798	0.17034	0.28666	0.34364
