# Supplementary material for: A Reconstructed Human Melanoma-in-Skin Model to Study Immune Modulatory and Angiogenic Mechanisms Facilitating Initial Melanoma Growth and Invasion
Source: Cancers (Basel). 2023 May 20;15(10):2849. doi: 10.3390/cancers15102849 (PMC10216824; doi:10.3390/cancers15102849)
Supplement: Supplementary file 1 [file cancers-15-02849-s001.zip › Table S3.pdf]

p value

	CD1a+	CD1a-CD14-	CD14+	BDCA3+	BDCA3-CD14-	BDCA3-CD14+	BDCA3+CD14-	BDCA3+CD14+	CD80+	CD80 MFI	CD163+	CD163 MFI	CD16+	CD16 MFI	CD14+CD163+CD16+	PD-L1+	PD-L1 MFI	PD-L2+	PD-L2 MFI
IL-6	0.566340	0.497208	0.705485	0.163711	0.226820	0.940852	0.970656	0.253647	0.329764	0.311409	0.787161	0.979386	0.681586	0.506113	0.526620	0.149104	0.074669	0.172146	0.032416
IL-8	0.599937	0.564227	0.739890	0.059851	0.199063	0.980599	0.939462	0.182013	0.462123	0.388216	0.549260	0.763992	0.697427	0.697846	0.694804	0.065855	0.024026	0.167916	0.017167
CXCL10	0.139219	0.751087	0.534977	0.382039	0.832484	0.718230	0.953138	0.585787	0.869414	0.437987	0.924267	0.561376	0.118981	0.180866	0.097945	0.259120	0.191265	0.297249	0.055466
CCL2	0.693415	0.373143	0.567059	0.004238	0.209313	0.568373	0.175441	0.003255	0.132623	0.061141	0.837639	0.912877	0.501435	0.246335	0.450047	0.216393	0.020589	0.588459	0.214266
CCL5	0.221260	0.975944	0.754002	0.086790	0.488875	0.750294	0.880305	0.269979	0.727195	0.366598	0.737691	0.930032	0.257846	0.436158	0.306669	0.066600	0.027180	0.192536	0.008709
VEGF	0.315963	0.759816	0.627339	0.610769	0.799957	0.964407	0.532920	0.948147	0.867101	0.860702	0.696745	0.788031	0.100095	0.071798	0.075714	0.282859	0.322844	0.246683	0.066847
IL-10	0.946256	0.982846	0.938422	0.932465	0.771562	0.702597	0.997989	0.758208	0.896934	0.669116	0.305868	0.168834	0.816738	0.575086	0.417391	0.187821	0.707109	0.544127	0.810669
TGFβ	0.115299	0.888780	0.609571	0.020666	0.699310	0.299653	0.407207	0.041224	0.578889	0.080649	0.521627	0.300938	0.287164	0.627054	0.234675	0.408574	0.031262	0.578935	0.045041
M-CSF	0.411972	0.413290	0.688034	0.043791	0.347541	0.466835	0.005039	0.013596	0.065102	0.015055	0.544995	0.740748	0.296428	0.018228	0.177926	0.244427	0.110815	0.623570	0.475130

Pearson *r* value

	CD1a+	CD1a-CD14-	CD14+	BDCA3+	BDCA3-CD14-	BDCA3-CD14+	BDCA3+CD14-	BDCA3+CD14+	CD80+	CD80 MFI	CD163+	CD163 MFI	CD16+	CD16 MFI	CD14+CD163+CD16+	PD-L1+	PD-L1 MFI	PD-L2+	PD-L2 MFI
IL-6	0.240385	-0.282885	0.159773	0.543634	-0.481683	0.031567	-0.015653	0.458116	0.397283	0.411271	0.114511	-0.010995	-0.173257	-0.277293	-0.264554	0.559732	0.660399	0.534693	0.749134
IL-8	0.220400	-0.241656	0.140566	0.686885	-0.507633	0.010348	0.032309	0.524527	0.305310	0.354978	0.250701	0.127238	-0.164305	-0.164069	-0.165784	0.675732	0.774536	0.539146	0.799806
CXCL10	0.571124	0.134362	-0.259416	0.359306	-0.089824	-0.152631	-0.025004	0.228770	0.069873	0.321136	-0.040435	-0.243372	-0.595941	-0.525694	-0.624416	0.453465	0.515260	0.422333	0.695459
CCL2	0.166567	-0.365594	0.239953	0.876953	-0.497843	-0.239165	0.531264	0.887632	0.578976	0.684434	-0.087031	0.046533	0.280226	0.464409	0.313185	0.491226	0.786540	0.227185	0.493201
CCL5	0.486743	0.012831	-0.132750	0.640958	-0.288154	-0.134800	0.064012	0.444381	0.147627	0.370264	0.141787	-0.037351	-0.454543	-0.322350	-0.414947	0.674391	0.764428	0.514005	0.842127
VEGF	0.407765	0.129540	-0.204372	0.214037	-0.107515	0.018988	-0.260678	0.027669	-0.071119	0.074568	0.164689	-0.114035	-0.621354	-0.665272	-0.658653	0.433838	0.402513	0.464107	0.673948
IL-10	-0.028679	-0.009149	0.032865	0.036050	-0.123071	0.161396	-0.001072	-0.130427	-0.055080	-0.180344	0.415570	0.538174	0.098372	0.235145	0.334930	0.518682	0.158861	0.253824	0.101675
TGFβ	-0.600705	-0.059457	0.214739	-0.786257	0.163245	0.420437	-0.341857	-0.726510	-0.232875	-0.650595	0.267639	0.419427	0.430367	0.204537	0.474642	-0.340923	-0.752367	-0.232847	-0.717625
M-CSF	0.338607	-0.337710	0.169607	0.720482	-0.384078	-0.302260	0.869391	0.815562	0.677097	0.808844	-0.253295	0.140090	0.422982	0.795528	0.528701	0.466066	0.606625	0.206563	0.296921
