# Supplementary figures and images for: A Reconstructed Human Melanoma-in-Skin Model to Study Immune Modulatory and Angiogenic Mechanisms Facilitating Initial Melanoma Growth and Invasion
Source: Cancers (Basel). 2023 May 20;15(10):2849. doi: 10.3390/cancers15102849 (PMC10216824; doi:10.3390/cancers15102849)

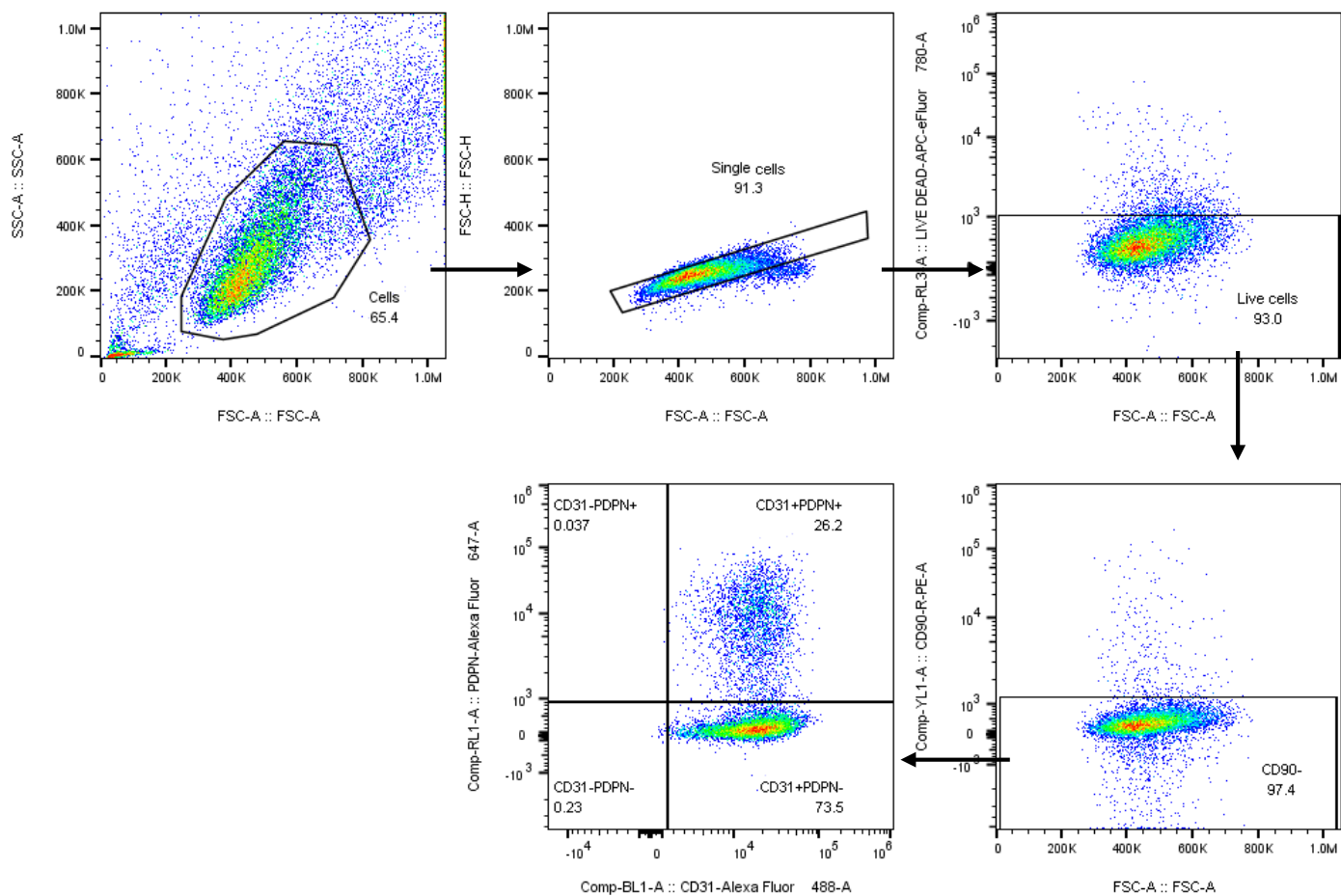

Supplement: Supplementary file 1 [file cancers-15-02849-s001.zip › Figure S1.pdf]

(a) pro-inflammation

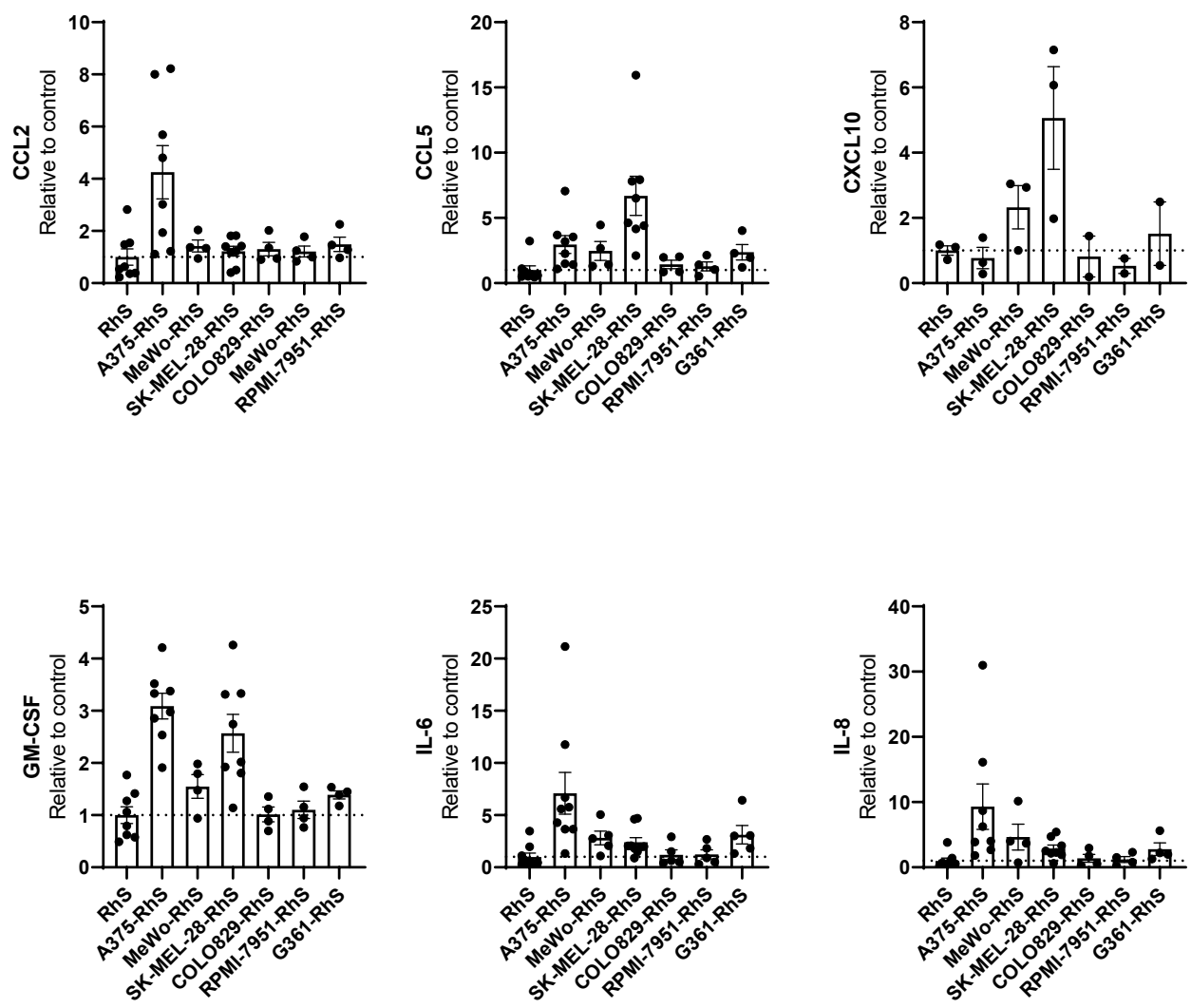

(b) anti-inflammation

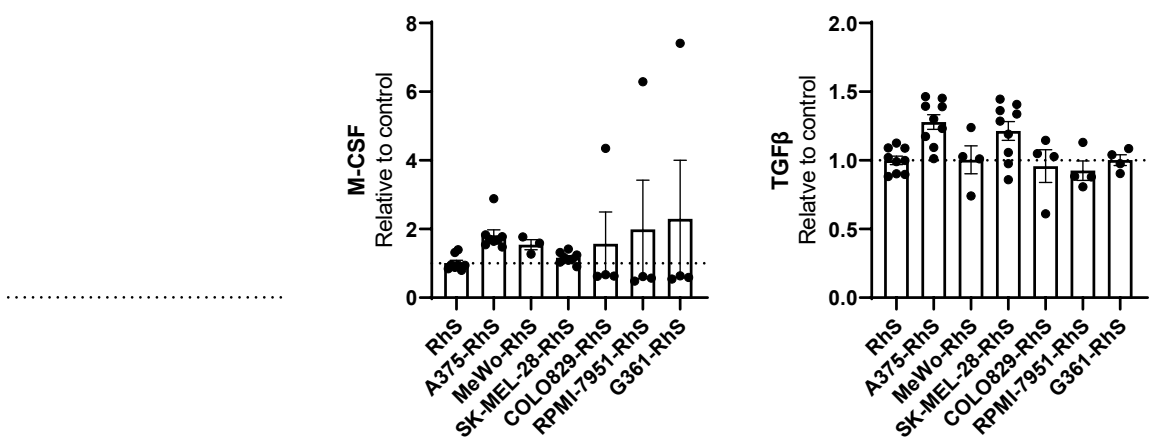

Supplement: Supplementary file 1 [file cancers-15-02849-s001.zip › Figure S2.pdf]

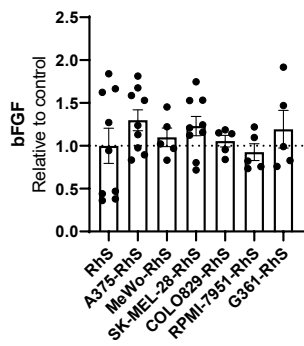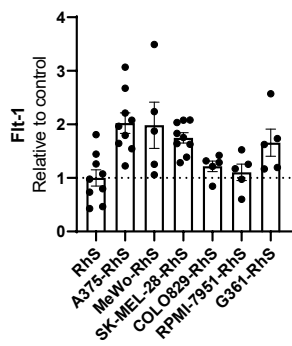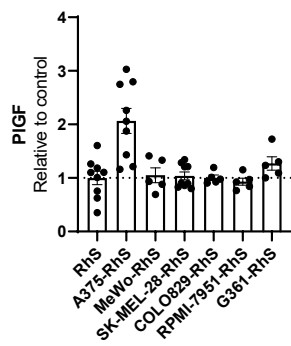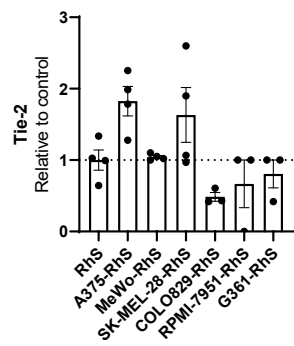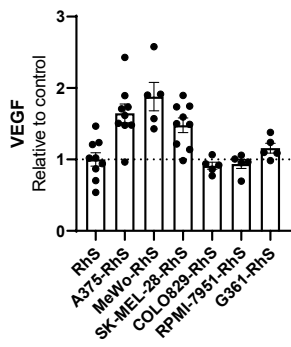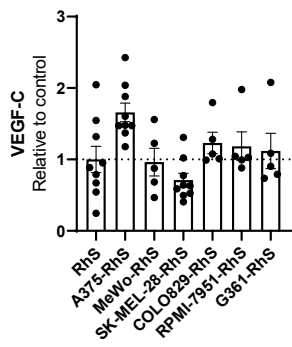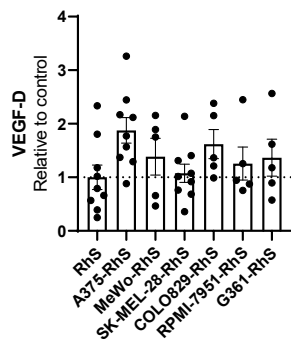

Supplement: Supplementary file 1 [file cancers-15-02849-s001.zip › Figure S3.pdf]

## 3D model

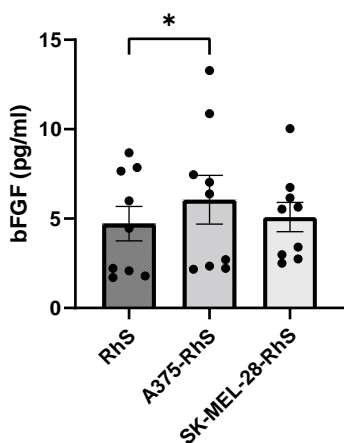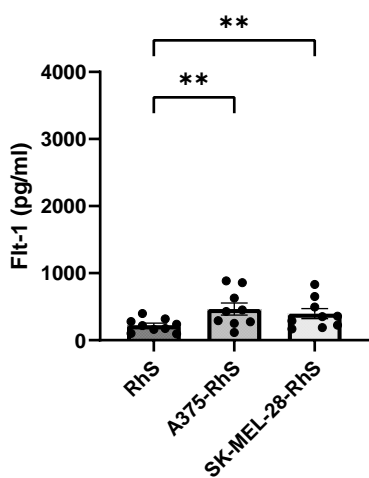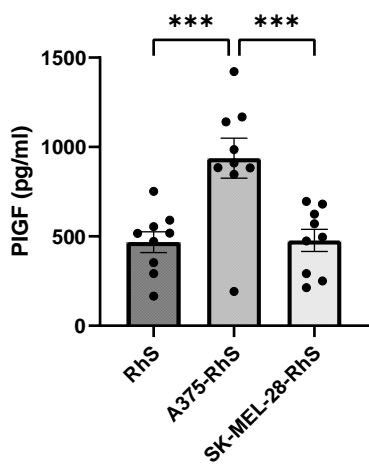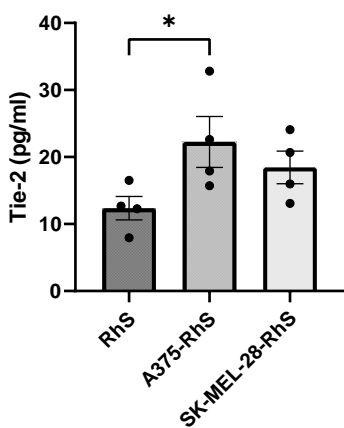

## 2D culture

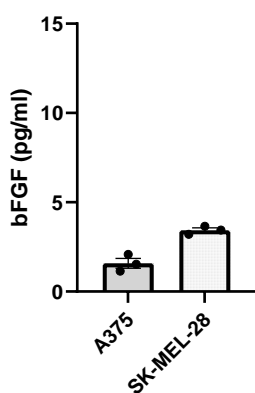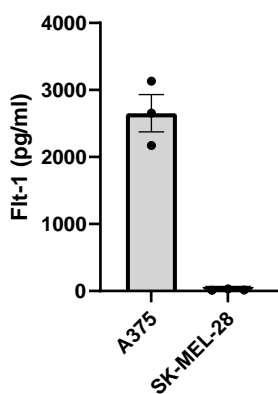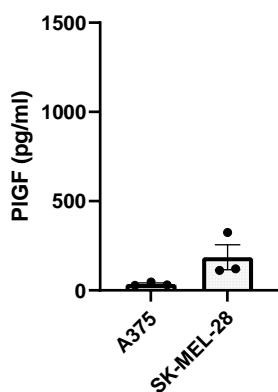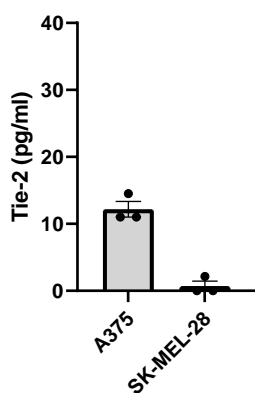

## 3D model

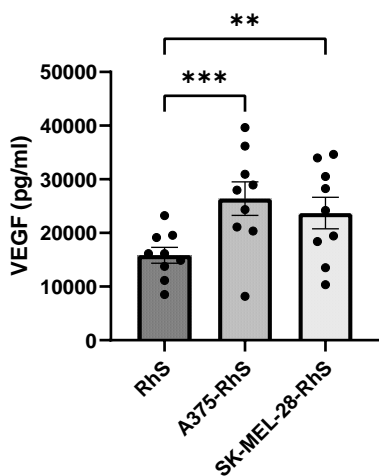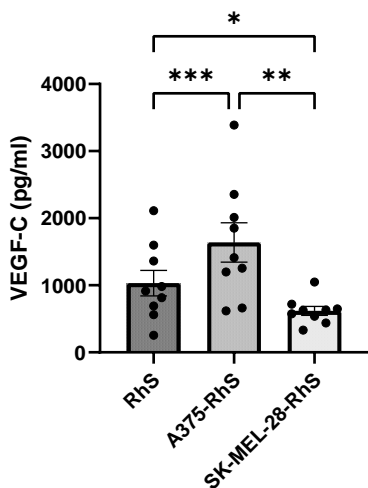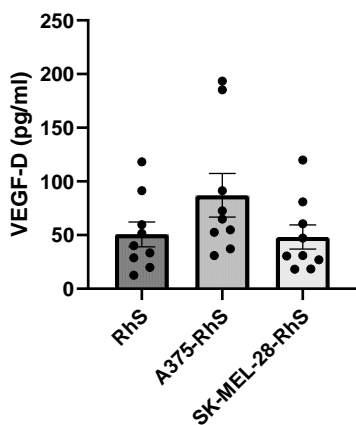

## 2D culture

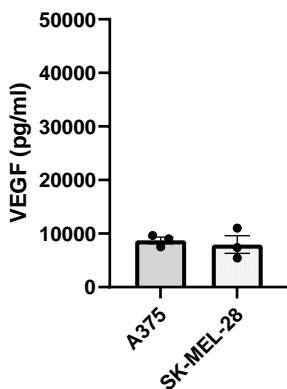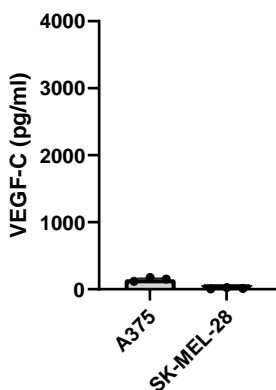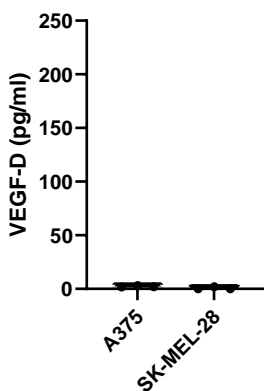

Supplement: Supplementary file 1 [file cancers-15-02849-s001.zip › Figure S4.pdf]

RPMI-7951-RhS

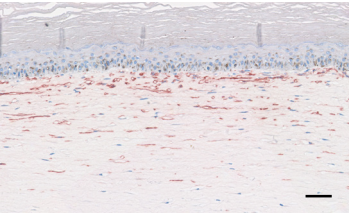

COLO829-RhS

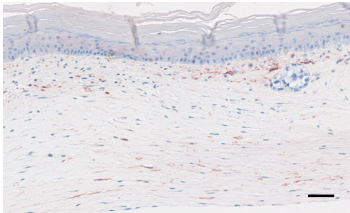

G361-RhS

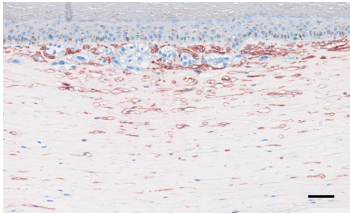

MeWo-RhS

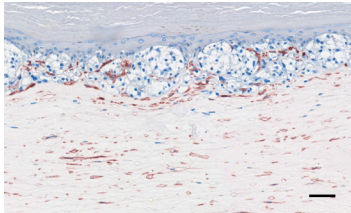

Supplement: Supplementary file 1 [file cancers-15-02849-s001.zip › Figure S5.pdf]
